# Simulations of dislocation contrast in dark-field X-ray microscopy

**DOI:** 10.1107/S1600576724001183

**Published:** 2024-03-21

**Authors:** Sina Borgi, Trygve Magnus Ræder, Mads Allerup Carlsen, Carsten Detlefs, Grethe Winther, Henning Friis Poulsen

**Affiliations:** a Department of Physics, Technical University of Denmark, 2800 Kongens Lyngby, Denmark; b European Synchrotron Radiation Facility, 71 avenue des Martyrs, CS40220, 38043 Grenoble Cedex 9, France; c Department of Civil and Mechanical Engineering, Technical University of Denmark, 2800 Kongens Lyngby, Denmark; Ecole National Supérieure des Mines, Saint-Etienne, France

**Keywords:** diffraction microstructure imaging, X-ray imaging, dark-field X-ray microscopy, geometrical optics, wavefront propagation

## Abstract

Dark-field X-ray microscopy (DFXM) is a full-field imaging technique that non-destructively maps the structure and local strain inside deeply embedded crystalline elements in three dimensions. In DFXM, an objective lens is placed along the diffracted beam to generate a magnified projection image of the local diffracted volume. This work explores contrast methods and optimizes the DFXM setup specifically for the case of mapping dislocations. Forward projections of detector images are generated using two complementary simulation tools based on geometrical optics and wavefront propagation, respectively. Weak and strong beam contrast and the mapping of strain components are studied. The feasibility of observing dislocations in a wall is elucidated as a function of the distance between neighbouring dislocations and the spatial resolution. Dislocation studies should be feasible with energy band widths of 10^−2^, of relevance for fourth-generation synchrotron and X-ray free-electron laser sources.

## Abstract 2

Forward projections of detector images are generated using two complementary simulation tools based on geometrical optics and wavefront propagation, respectively. The feasibility of observing dislocations in a domain wall is elucidated as a function of different instrumental settings, scan modes, dislocation densities in the wall and spatial resolution.

## 1. Introduction

Dark-field X-ray microscopy (DFXM) is a novel full-field imaging technique that non-destructively maps the 3D structure, orientation and strain fields of deeply embedded crystalline elements such as grains or domains (Simons *et al.*, 2015 ▸; Poulsen *et al.*, 2017 ▸; Poulsen, 2020 ▸). Direct-space images are formed by placing an X-ray objective lens along the diffracted beam. The magnification and field of view (FOV) can be adjusted by changing the optics configuration, with a limit on the spatial resolution of currently 100 nm. On beamline ID06 of the European Synchrotron Radiation Facility (Kutsal *et al.*, 2019 ▸), DFXM has been used to study domain evolution in *e.g.* ferroelectrics (Simons *et al.*, 2018 ▸), shape-memory alloys (Bucsek *et al.*, 2019 ▸), metals (Mavrikakis *et al.*, 2019 ▸; Ahl *et al.*, 2020 ▸; Hlushko *et al.*, 2020 ▸; Dresselhaus-Marais *et al.*, 2021 ▸; Zelenika *et al.*, 2023 ▸) and biominerals (Cook *et al.*, 2018 ▸). In 2019 the method was transferred to use with an X-ray free-electron laser (XFEL) (Dresselhaus-Marais *et al.*, 2023 ▸), an approach subsequently applied to image the propagation of sound waves in diamond (Holstad *et al.*, 2023 ▸).

DFXM is conceptually similar to dark-field electron microscopy in transmission electron microscopy (TEM), which is used extensively to image local orientation and strain (Williams & Carter, 1996 ▸; Nellist, 2000 ▸). In TEM, forward modelling is often an essential part of the data analysis chain (Vulovic *et al.*, 2013 ▸).

We believe that a similar effort in forward modelling is required in DFXM to understand contrast, to optimize experiments and as part of training and, in particular, to enable direct coupling to materials and thermomechanical modelling.

To facilitate this, a geometrical optics formalism for forward modelling of DFXM images based on micromechanical models was presented by Poulsen *et al.* (2021 ▸). There the deformation was expressed in terms of a deformation gradient tensor field, 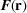 , with **r** symbolizing position in space. Analytical expressions were provided for simplified cases. This was supplemented by the presentation of Monte Carlo (MC) code for sampling the 6D direct space–reciprocal space instrumental resolution function Res and for constructing the forward model itself. Note that the reciprocal-space part of the resolution function is typically highly anisotropic (Poulsen *et al.*, 2018 ▸), a fact that has a strong effect on contrast mechanisms and which can be used to simplify the simulations.

A complementary wavefront propagation simulation tool was presented by Carlsen *et al.* (2022 ▸). In this, numerical simulations of image formation were performed by numerical integration of the dynamical Takagi–Taupin equations (Takagi, 1962 ▸, 1969 ▸; Taupin, 1967 ▸) and wavefront propagation. This tool was validated by comparing simulated images with experimental data from a near-perfect single crystal of diamond containing a single stacking-fault defect in the illuminated volume.

In this paper we will focus on the prospects of DFXM for multiscale visualization of individual dislocations and networks of dislocations (Jakobsen *et al.*, 2019 ▸; Porz *et al.*, 2021 ▸; Dresselhaus-Marais *et al.*, 2021 ▸; Yildirim *et al.*, 2023 ▸). We first compare the results of both geometrical optics and wavefront simulations for idealized dislocation boundaries in order to discuss applicability, while at the same time prime contrast mechanisms are illustrated by both simulation tools. Specifically, we simulate DFXM images of edge dislocations in single-crystal aluminium, using an experimental setup that is identical to the synchrotron experiment presented by Dresselhaus-Marais *et al.* (2021 ▸).

Next we address several issues of key relevance for the design of experiments, such as spatial resolution and the suitability of using a beam with a relative energy band width of 10^−3^ or 10^−2^, of relevance to fourth-generation synchrotron sources and XFELs. We also present a heuristic solution for how to extract a map of (components of) the field 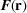 from the forward simulated images, thereby closing the loop from a micromechanical model of a dislocation configuration to DFXM images and back.

## 2. Experimental

### 2.1. Microscopy principles

**Figure 1:**
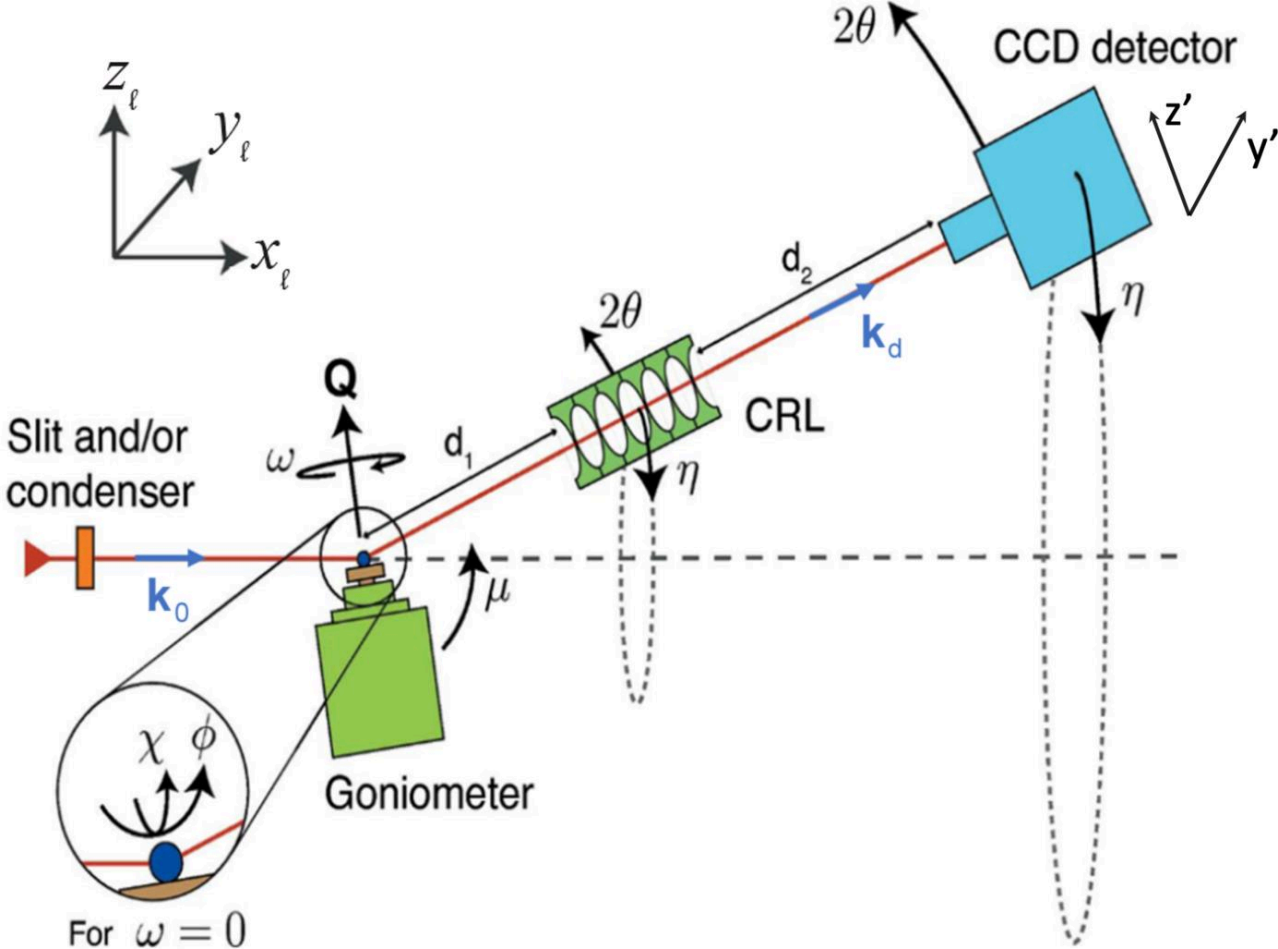
The geometry of the dark-field X-ray microscope on ID06 at the ESRF, shown in the laboratory coordinate system, (*x*_l_, *y*_l_, *z*_l_). The incident beam is defined by the wavevector **k**_0_, while the diffracted beam is defined by the wavevector **k**_d_. The pivot point of the goniometer and sample is coincident with the intersection of the two optical axes. The vector **Q** defines the local scattering vector at a given point (*x*, *y*, *z*) within the sample, and may be parameterized by the scattering angle 2θ, the azimuthal angle η and the length of the vector |**Q**|. The value of |**Q**| is related to the spacing of the lattice plane being measured *d*_*hkl*_ and the X-ray wavelength λ by Bragg’s law. The goniometer is associated with a base tilt μ, an ω rotation around **Q** and two tilts, χ and ϕ. *d*_1_ is the distance from the sample to the entry point of the objective and *d*_2_ is the distance from the exit point of the objective to the detector. CRL denotes the compound refractive lens. This figure is adapted from Poulsen *et al.* (2017 ▸).

The geometry of DFXM was presented in detail by Poulsen *et al.* (2017 ▸). The layout is illustrated in Fig. 1 ▸ along with the laboratory coordinate system (*x*_l_, *y*_l_, *z*_l_). A nearly monochromatic and nearly collimated X-ray beam with an average energy *E* illuminates the sample. This beam may be condensed in the vertical and horizontal directions to generate a beam with a divergence that has dimensions of Δζ_v_ and Δζ_h_, respectively. In the following we shall assume that the vertical condensation enables the creation of a line beam impinging on the sample, characterized by a sub-micrometre vertical beam height Δ*z*_l_.

The goniometer is designed to access diffraction angles in a nearly vertical scattering geometry and to probe reciprocal space only in the immediate vicinity of a given reflection *hkl*. In the implementation of DFXM on ID06 at ESRF, this was achieved by rotating the sample by a combination of the μ, ω, χ and ϕ rotation stages (Fig. 1 ▸). The direction of the diffracted beam is characterized by the scattering angle 2θ and the azimuthal angle η.

Following Poulsen *et al.* (2021 ▸), we can for the purpose of these simulations consider a simplified geometry characterized by perfect alignment, μ = θ_0_, ω = 0 (fixed), η = 0 and 2θ = 2θ_0_ for the nominal *hkl* reflection. We assume that during the experiment, ϕ is scanned (‘rocked’) over a small range centred around ϕ = 0 and/or χ is scanned (‘rolled’) over a small range centred around χ = 0 and/or 2θ is changed over a small range centred around 2θ = 2θ_0_. This last scan implies a movement of the ‘2θ arm’ comprising both the objective and the detector.

The optical axis of an X-ray objective is aligned to the diffracted beam for the nominal *hkl* value to produce a magnified image (inverted in both directions) on the 2D detector. The key attributes of the objective that are important to this study are the numerical aperture NA and the focal distance *f*_*N*_. The position and tilt of this objective define the primary imaging system in DFXM, which is associated with an *object plane* inside the sample (at the pivot point of the goniometer, Fig. 1 ▸) and an *image plane* coinciding with the plane of the detector. In the line beam configuration of this paper, the illuminated plane is effectively projected onto the detector at an angle of 2θ. The distance *d*_1_ spans from the object plane to the entry point of the objective and *d*_2_ is the distance from the exit point of the objective to the detector. The image generated by the objective has an associated magnification 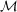 and FOV and can be subject to vignetting and depth of focus issues (Poulsen *et al.*, 2017 ▸).

During a range of small tilts around the nominal position, the diffraction condition of the undeformed crystal lattice is met. This is defined as the *strong beam* condition. During the simulations, the rotating stages will bring the sample slightly outside this diffraction condition, while bringing the dislocation strain fields into the diffraction condition, and this is defined as the *weak beam* condition.

### 2.2. Digital test objects

**Figure 2:**
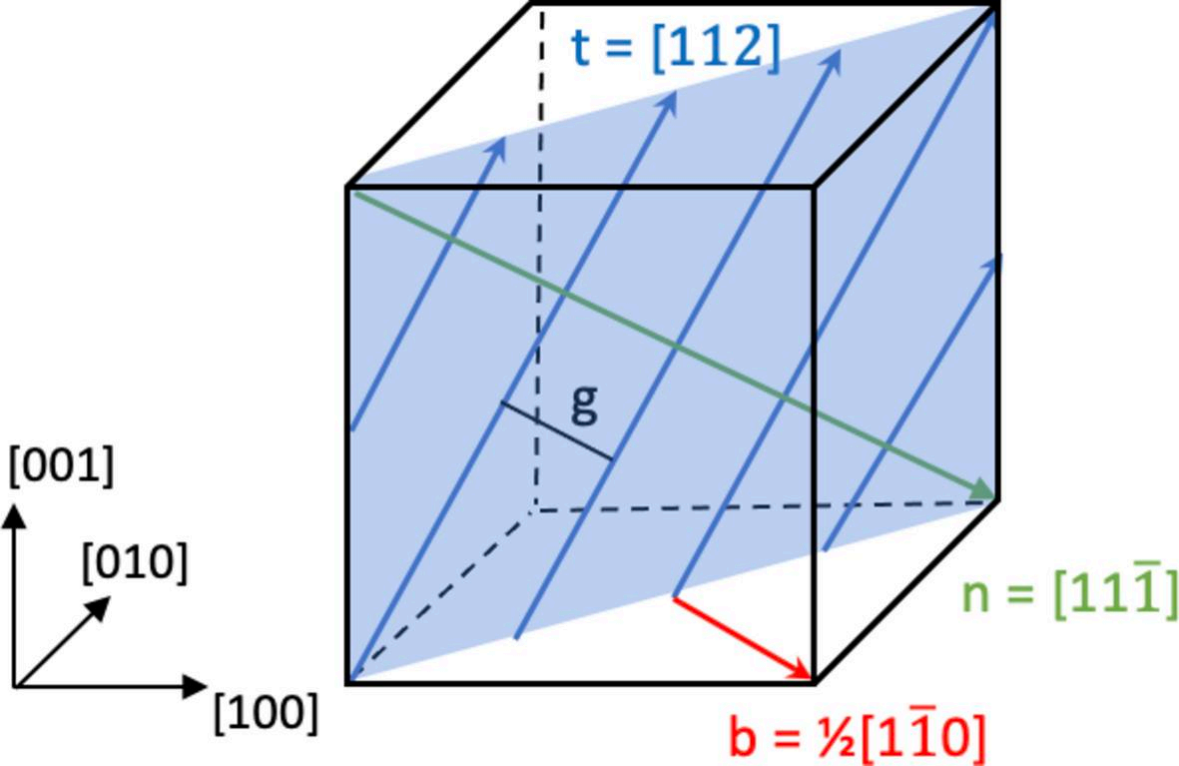
The phantom used and its relation to the grain coordinate system (shown to the left). The tilt boundary on the 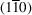 plane (in blue) consists of straight edge dislocations, all with Burgers vector **b**, line direction **t** and glide plane normal **n** in an f.c.c. crystal lattice. *g* is the distance between neighbouring dislocations. See the text for further discussion.

In this work the phantom or digital test object we shall study is a simple tilted wall of straight edge dislocations. A dislocation is defined by its Burgers vector **b**, its line direction **t** and the normal to its glide plane **n**. In a face-centred cubic (f.c.c.) lattice, these directions align with 〈110〉, 〈112〉 and 〈111〉, respectively. Note that dislocations with the same Burgers vector but of opposite sign will have oppositely directed line vectors, which reverses the sign of the displacement field around the dislocation. For this work we selected one of the 12 symmetry-related systems, the one with **b** = 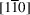 , **n** = [112] and **t** = 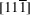 , as illustrated in Fig. 2 ▸. Associated with this we define an orthonormal *dislocation coordinate system* by 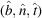 , as also shown in the figure. We identify this by the subscript d. For reference, we provide analytical expressions for the displacement field and the corresponding deformation gradient tensor field 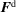 for one edge dislocation in this coordinate system in Appendix *A* [App appa]. For simplicity, we employ isotropic elasticity and use aluminium as the model material. However, the methodology may be extended to include elastic anisotropy. As illustrated in Fig. 2 ▸, we position identical dis­locations of this type displaced from each other in direction **n**.

This configuration forms a low-angle grain boundary normal to the Burgers vector, with a tilt of the lattices about the line direction and of magnitude α = |**b**|/*g*, where *g* is the distance between the individual dislocations measured along the direction of **n**. It is well known that an infinite wall of edge dislocations does not exhibit long-range stress fields (Hull & Bacon, 2011 ▸). Here we have added dislocations to the wall outside the FOV until end effects were avoided, *i.e.* the wall may be considered infinite for all subsequent simulations.

### 2.3. Diffraction formalism

Poulsen *et al.* (2021 ▸) presented a formalism for diffraction whereby a micromechanical model can be forward projected to DFXM images. Following this formalism we define a *grain coordinate system*, with unit axes 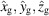 collinear with [100], [010] and [001], respectively (see Fig. 2 ▸). The rotation matrix **U**_d_ that transforms a direct-space vector **r** or a reciprocal-space vector **Q** from the dislocation system into this grain system is given by 

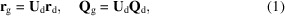




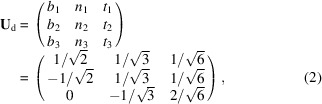

where the columns of **U**_d_ are the components of the normalized vectors **b**, **n** and **t**.

**Figure 3:**
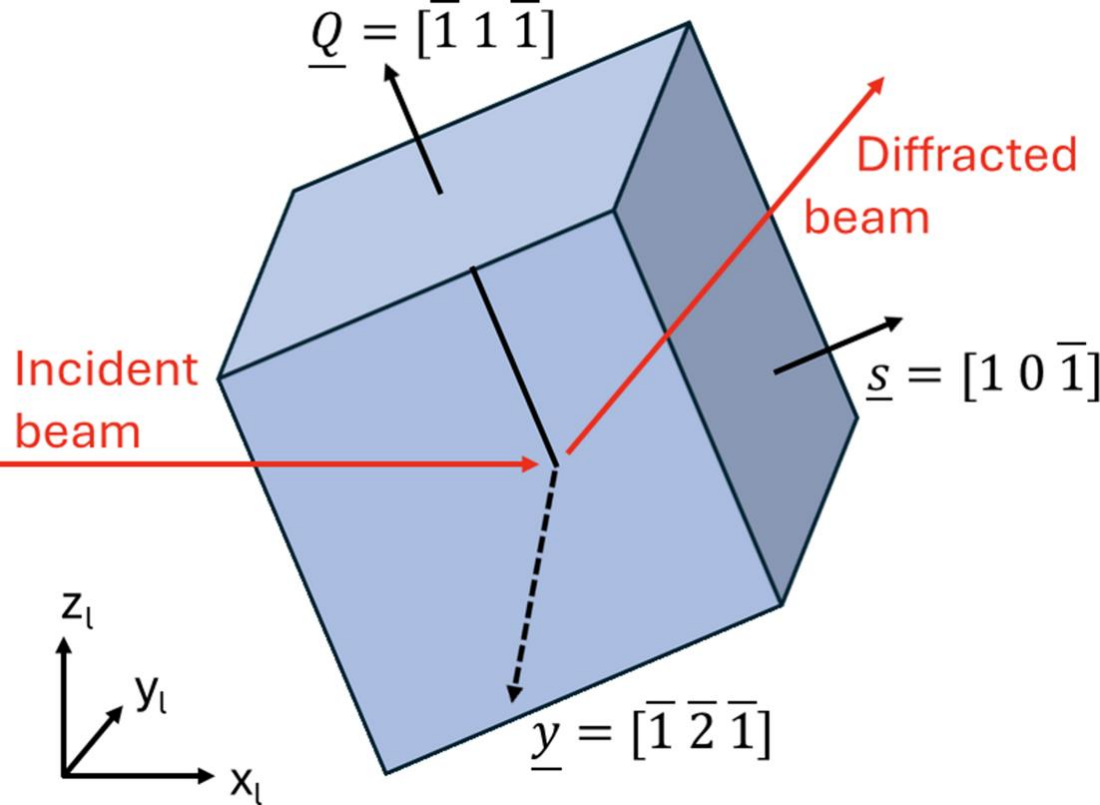
The sample system (blue box) with axes (**s**, **y**, **Q**) given by coordinates in the grain system. Also shown is the laboratory system (*x*_l_, *y*_l_, *z*_l_), rotated with respect to the sample coordinate system by an angle of θ around the common *y* axis. A symmetric geometry is defined with diffraction vector **Q** = 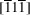 , (*x*_l_, *z*_l_) being the scattering plane and the sample surface normal being along 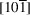 .

Next we introduce a *sample coordinate system*, identified by the subscript s. To ease the two-beam simulations we choose a symmetric scattering geometry with a 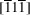 scattering vector and scattering within the (*x*_l_, *z*_l_) plane. As illustrated in Fig. 3 ▸, the sample is a slab with surface normal **s** = 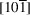 . The sample is aligned such that the *y* axis of the laboratory coordinate system *y*_l_ is parallel to the crystal direction 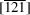 . This geometry implies a rotation matrix from the sample to the grain coordinate system by 

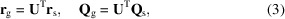




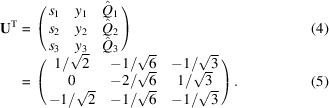




The sample system is related to the laboratory frame by a rotation of −θ about the *y* axis, 

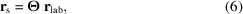




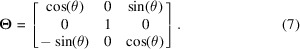

So all in all, the laboratory frame coordinates are related to the dislocation frame coordinates by 

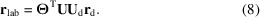




### 2.4. Specifics of the microscopy setup

The default geometry in the simulations is similar to that of the experiment by Dresselhaus-Marais *et al.* (2021 ▸) and the simulations by Poulsen *et al.* (2021 ▸). Specifically, the sample is illuminated with a 17.00 keV beam with an energy band width (FWHM) of Δ*E*/*E* = 1.41 × 10^−4^. The use of a condenser implies that the incident beam has a vertical divergence with a truncated Gaussian profile. The attenuation through the condenser lens gives rise to a Gaussian with a width (FWHM) of Δζ_v_ = 0.53 mrad, while the physical aperture of the condenser gives rise to a hard cutoff at ±140 µrad. The corresponding vertical intensity profile of this incident beam is a Gaussian with an FWHM of 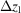 = 0.15 µm (Carlsen *et al.*, 2022 ▸). In the *y*_l_ direction the beam is assumed to be parallel and homogeneous across our FOV. With diffraction from a {111} reflection, the scattering angle becomes 2θ_0_ = 17.953°.

The objective is a compound refractive lens (CRL; Snigirev *et al.*, 2009 ▸) with *N* = 88 Be lenses, each with a radius of curvature of *R* = 50 µm, a physical aperture of *D* = 0.566 mm and a distance between the centres of neighbouring lenses of *T* = 1.6 mm. With geometrical optics we have analytical expressions for the imaging geometry and acceptance function of the objective (Poulsen *et al.*, 2017 ▸). The results are a focal length of *f*_*N*_ = 192 mm, an X-ray magnification 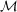 = 16.85 and a numerical aperture (FWHM) of NA = 0.731 mrad.

The 2D detector is placed on a vertical translation stage positioned at *x* = 4778 mm downstream from the detector, giving a total length of the optical path from the centre of rotation to the detector screen of 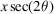 = 5023 mm. The resulting effective pixel sizes, as measured in the (*x*_l_, *y*_l_) plane, are 123.45 and 40.00 nm in the *x*_l_ and *y*_l_ directions, respectively.

### 2.5. Geometrical optics simulations

The forward projection algorithm and experimental setup used are identical to those presented by Poulsen *et al.* (2021 ▸) ▸ in the ‘simplified geometry’. The simulation code is valid for all crystal systems and all reflections. The relevant equations for associating an **F** field with positions in DFXM images are 

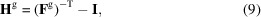




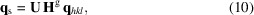




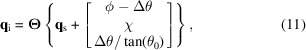

with i referring to the imaging coordinate system.

Initially the reciprocal-space resolution function Res_*q*_ is estimated by Monte Carlo simulations and found to be a highly anisotropic disc with the thin direction along the rocking direction and the diameter of the plate equal to NA [Fig. 3 of Poulsen *et al.* (2021 ▸)]. Next, the forward model for simulation of DFXM images is constructed on the basis of equation (58) in the same paper and using Res_*q*_ as a look-up table. The intensity of the simulations involves modelling how the diffraction from the crystal interacts with the resolution function of the experimental setup. This interaction is described mathematically by integrating the product of the diffraction intensity and the resolution function over both real and reciprocal space. By defining the integration volume on the basis of the pixel dimensions, the resulting integrated intensity represents what is recorded on a specific pixel of the detector in a DFXM image, as described by Poulsen *et al.* (2021 ▸).

By default, the intensities are normalized to experimental data. Specifically, the average intensity at the nominal position in reciprocal space (and far from any dislocations in direct space) is set to 100 counts per pixel on the detector. The data are subject to Poisson noise, while background, read-out noise and other potential noise terms are neglected. The contrast in most images presented is displayed with the same colour scale for direct comparison.

### 2.6. Wavefront propagation simulations

Carlsen *et al.* (2022 ▸) presented numerical simulations of DFXM image formation using integration of the dynamical Takagi–Taupin equations and wavefront propagation. The simulations involve propagation through both the sample and the thick lens objective to the detector, and as such they fully integrate coherence and dynamical diffraction effects. The approach was validated by comparing simulated images with experimental data from a diamond crystal containing a single stacking-fault defect in the illuminated volume. We here apply this approach for direct comparison with the geometrical optics results. Just as for the geometrical optics case described above, there is no noise introduced and, in the case of a series of images, the intensities are normalized to that of the strong beam condition.

Initially, the simulated microscope is focused by optimization based on wavefront propagation using the fractional Fourier transform approach outlined by Pedersen *et al.* (2018 ▸). The resulting X-ray magnification and working distance from the sample to the first lens in the CRL are 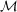 = 16.85 and *d*_1_ = 207.0 mm, respectively – nearly identical to the analytical results from geometrical optics.

### 2.7. The *q* map and centre-of-mass map

For an idealized instrument where the reciprocal-space resolution function is a delta function, and assuming the simplified geometry conditions, then varying the tilt angle ϕ corresponds to probing one direction in reciprocal space **q**_i,1_ (Poulsen *et al.*, 2021 ▸). Likewise, χ and 2θ scans correspond to probing **q**_i,2_ and **q**_i,3_, respectively. The normalized reciprocal-space vector **q**_i_ is linearly related to components of the **H** field, *cf*. equations (9) to (11) above.

Hence, it is natural to seek to condense the information in a set of (experimental or simulated) DFXM images generated for a series of ϕ values into a map of **q**_i,1_, where each pixel (corresponding to one voxel in the illuminated layer of the sample) is represented by one strain value. Likewise, to represent χ and 2θ scans in terms of **q**_i,2_ and **q**_i,3_ maps.

In reality DFXM images are strongly influenced by instrumental effects in several ways:

(i) *The singularity.* The spatial dependence of all tensor components (for screw or edge dislocations alike) exhibits inversion symmetry and varies with distance to the core, *r*, as 1/*r*. The fact that the microscope has a finite direct-space resolution implies that regions close to the core of a dis­location will give rise to contrast in images over a range of ϕ values (and the same for χ and 2θ).

(ii) *The comparability of the measured strains and the resolution function.* The maximum strain value ε at a distance *r* from a single dislocation line is ε_max_ ≃ *b*/(4π*r*), where *b* is the magnitude of the Burgers vector. With an in-plane resolution of 100 nm and *b* ≃ 0.3 nm, it appears that the maximum strain will be ε_max_ = 0.3/(4π × 50) = 4 × 10^−4^. As we shall explore in detail below, this is comparable to the angular resolution in the two directions where the angular resolution function is dominated by the numerical aperture of the objective.

One application of the forward projection formalism is for a given **F** field to simulate a set of DFXM images, compare these voxel by voxel with experimental data, derive a corresponding measure of the correlation, and then, in an iterative fashion, vary the assignment of **F** in individual voxels and optimize the correlation. Such a fitting algorithm would be a standard way of solving the inverse problem but it is computationally heavy.

As an alternative, we present a simpler algorithm, where (components of) **q**_i_ are determined directly from DFXM images. Noting that convolution with a symmetric function conserves the centre of mass (COM), we specifically propose to use a COM map. For a (ϕ, χ) scan the procedure is as follows:

(i) For each voxel in the sample the (ϕ, χ) intensity map is derived from the DFXM data.

(ii) The COM coordinates in this (ϕ, χ) intensity map are extracted, corresponding to **q**_i,1_ and **q**_i,2_, respectively.

The COM map has regularly been used to map domains, *e.g.* using the *darfix* data analysis software developed specifically for the handling of DFXM data at ESRF (Garriga Ferrer *et al.*, 2023 ▸). We here propose that this procedure may also be relevant for identifying dislocations. A second map of similar nature is the FWHM map, which associates each voxel with the width of the intensity distributions in ϕ, χ and/or 2θ. The latter gives information on the local disorder, *e.g.* dislocation densities.

## 3. Results

By default all simulations were performed at a constant incoming flux to make the contrast directly comparable. In the following the simulated DFXM images are not shown in detector coordinates. Instead, the images are shown in terms of laboratory coordinates (*x*_l_, *y*_l_) in the illuminated plane in the sample.

### 3.1. Strong and weak beam contrast in rocking scans

**Figure 4:**
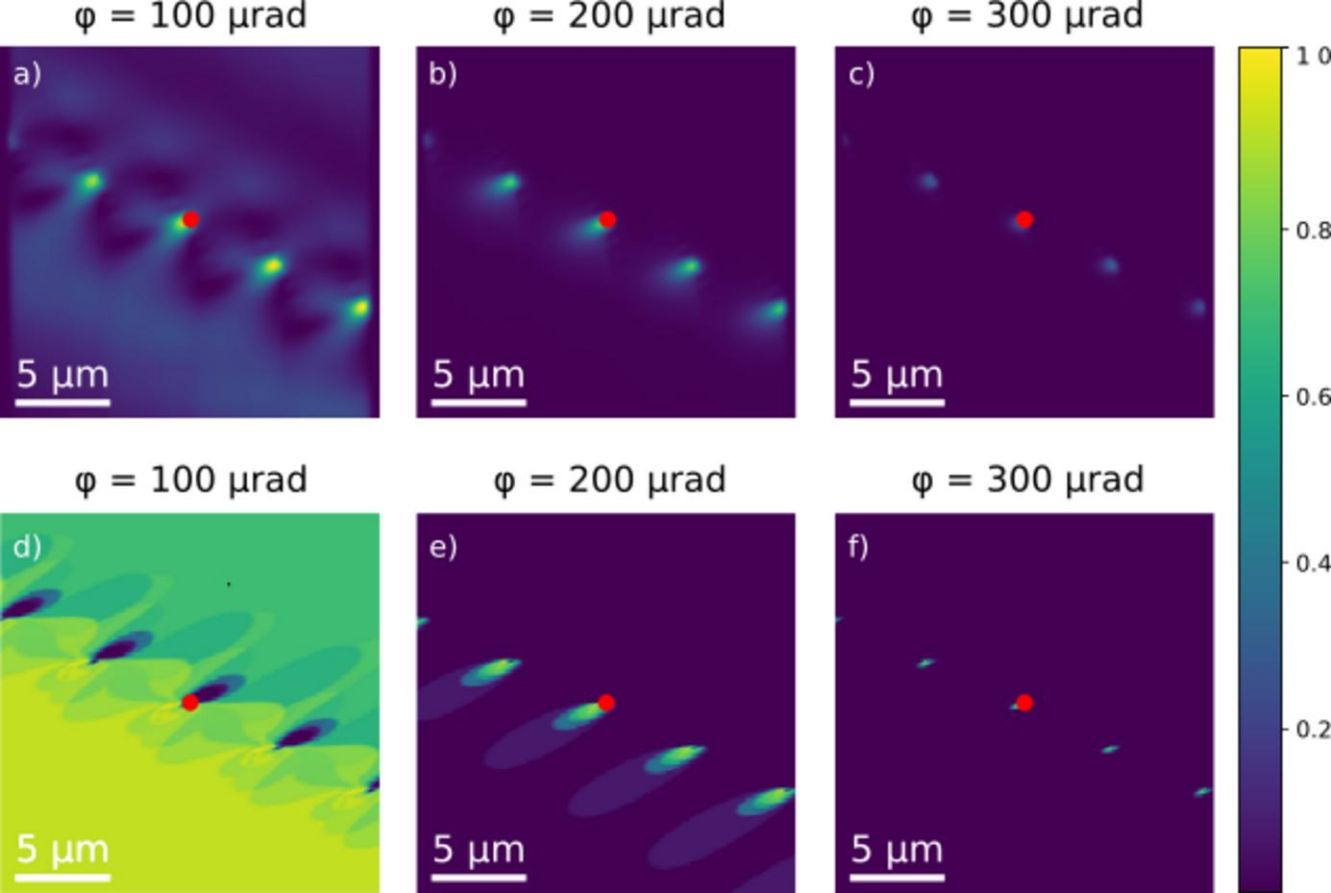
Sample-space projections of simulations of DFXM images for a phantom comprising a wall of edge dislocations in aluminium with 4 µm between the dislocation lines (Fig. 2). The position of the core of the central dislocation is indicated with a red dot. The tilt angle ϕ is varied from 100 (strong beam condition) to 300 µrad (weak beam condition). (*a*)–(*c*) Wavefront propagation results, displaying a region of interest of the illuminated sample, with varying tilt angles from 100 to 300 µrad. (*d*)–(*f*) Geometrical optics results for direct comparison with panels (*a*)–(*c*). All images are normalized to the maximum intensity of the strong beam condition (ϕ = 0).

Results from geometrical optics and wavefront propagation simulations are directly compared in Fig. 4 ▸, illustrating the contrast obtained during a rocking scan (a scan of the tilt angle ϕ). In the strong beam case, in Fig. 4 ▸(*a*) we observe the expected signatures for dynamical diffraction in the wavefront simulations, *e.g.* *Pendellösung* fringes near the entry surface. However, these effects seemingly do not disturb the contrast in the vicinity of the dislocations in a detrimental way. For increasing absolute values of ϕ, the contrast appears more and more localized in a region close to the core of a dislocation. Simultaneously, the geometrical optics and wavefront propagation simulations become nearly identical, as seen in Figs. 4 ▸(*b*)–4 ▸(*c*) and Figs. 4 ▸(*e*)–4 ▸(*f*). This demonstrates the merit of weak beam contrast and the validity of the geometrical optics approach in this limit. For a given exposure time, the maximum ϕ value at which the dislocation is still visible constitutes a limit on how close to the core we can probe the field.

Somewhat arbitrarily, we define the contrast limit by an average local intensity of 1. In these simulations the limit is around 1700 µrad, implying a shear strain field of 1.7 × 10^−3^. Additional simulations reveal that factors of 10 and 100 in the counting statistics improve this limit to 2.7 × 10^−3^ and 2.9 × 10^−3^, respectively. Note that these strain values correspond to regions with radii of approximately 14, 9 and 8 nm, respectively. Hence, it appears that we can detect regions that are substantially smaller than the in-plane spatial resolution (defined as the smallest distance between two identical objects at which they can still be distinguished from each other).

### 3.2. Strong and weak beam contrast in rolling and 2θ scans

**Figure 5:**
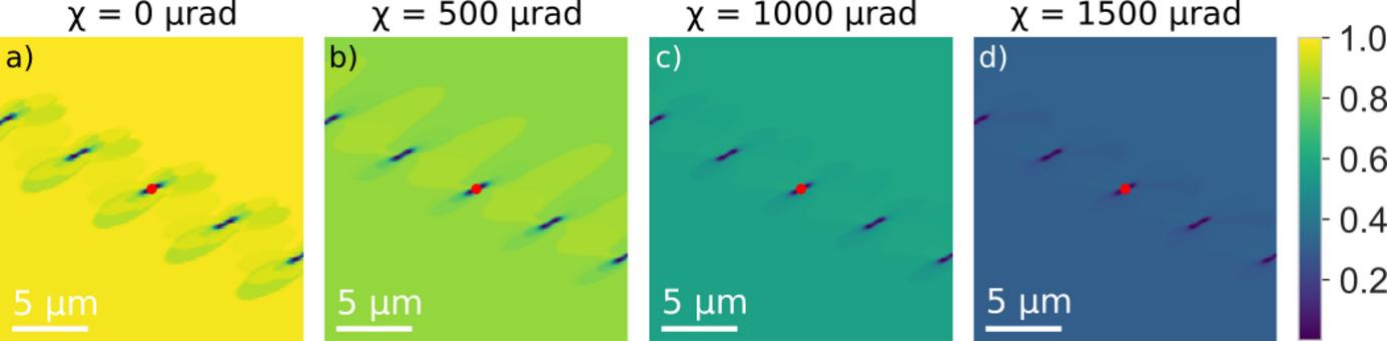
Geometrical optics simulations of DFXM images for the same phantom as used in Fig. 4 but now as a function of varying tilt angle χ from 0 (strong beam condition) to 1500 µrad (weak beam condition). The position of the core of the central dislocation is indicated with a red dot.

**Figure 6:**
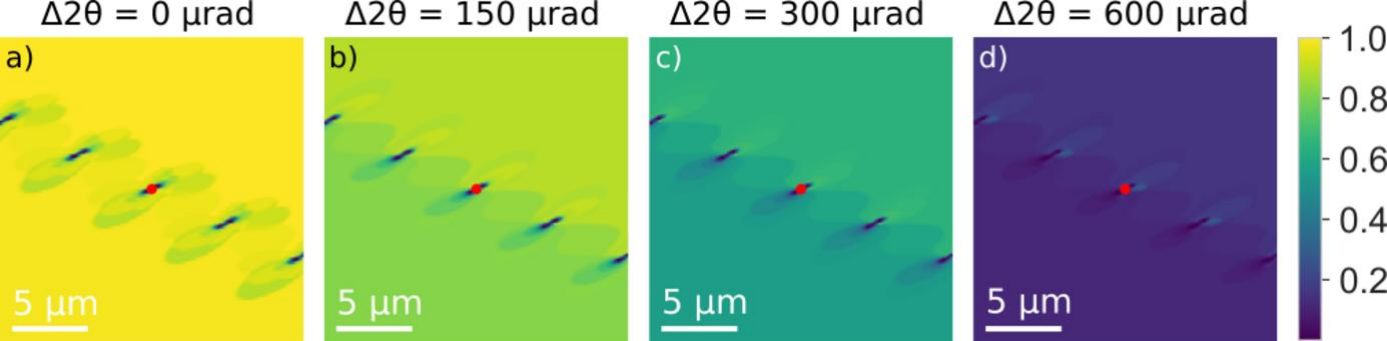
Geometrical optics simulations of DFXM images for the same phantom as used in Fig. 4 but now as a function of varying the scattering angle 2θ from the strong beam condition (Δ2θ = 0) to the weak beam condition ( 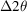 = 600 µrad). The position of the core of the central dislocation is indicated with a red dot.

The corresponding contrast for a rolling scan (for ϕ = 0) is shown in Fig. 5 ▸. In comparison with the rocking scan the reciprocal-space function is in this case dominated by the NA of the objective and does therefore extend beyond 1 mrad. This is substantially larger than the strain values we can observe (Section 3.1). Hence, the images are dominated by the strong beam contrast throughout. Apart from an overall decrease in intensity, the variation in contrast with χ is rather small. The corresponding contrast for a 2θ scan (for ϕ = χ = 0) is shown in Fig. 6 ▸. Again, in comparison with the rocking scan the reciprocal-space function is in this case dominated by the NA of the objective.

### 3.3. Effect of the vertical beam height

**Figure 7:**
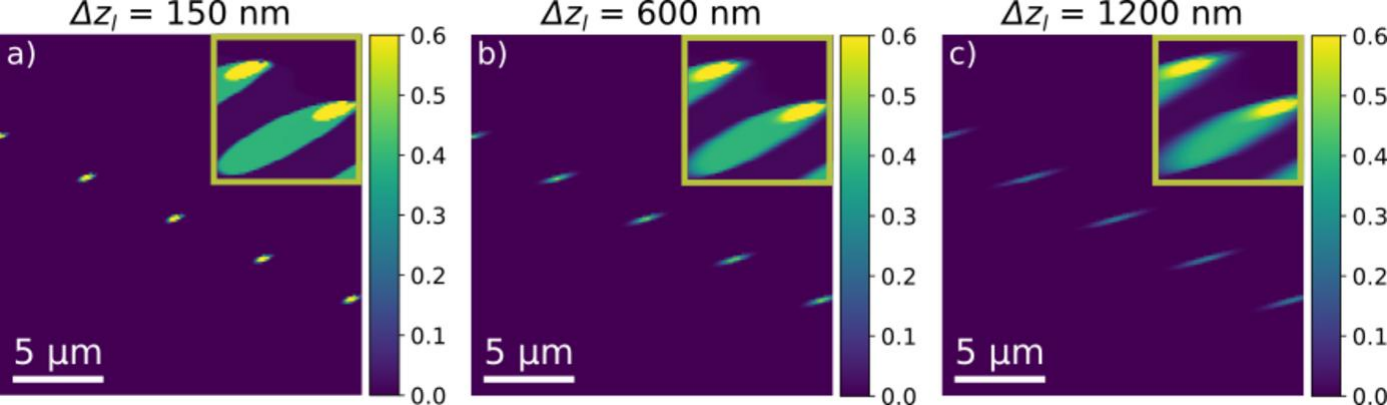
Forward images using ϕ = 300 µrad for a weak beam in a rocking curve to display the dislocation core with ϕ = 200 µrad for (insets) illuminating the strain field. Beam heights of (*a*) 150 nm, (*b*) 600 nm and (*c*) 1200 nm. The colour bar of the images is cut to 50% of the maximum strong beam intensity.

The diffraction limit implies that the incident beam cannot at the same time be infinitely small (Δ*z*_l_ → 0) and have zero divergence (Δζ_v_ → 0). With the existing type of condensers the minimum vertical beam height is around 150 nm, and this is the number used as the default in this work. However, in practice the experimentally determined beam height is substantially larger. To understand the effect on contrast, in Fig. 7 ▸ we show weak beam images acquired with Δ*z*_l_ = 600 nm and Δ*z*_l_ = 1200 nm. Comparing Figs. 7 ▸(*b*) and 7 ▸(*c*) with the Δ*z*_l_ = 150 nm data shown in Fig. 7 ▸(*a*), we see that, with increasing height, streaks appear in the direction of the projection of the dislocation line on the sample plane. The length of this streak (defined by the FWHM) can be substantial, *e.g.* 1.5 µm for Δ*z*_l_ = 600 nm and 2.5 µm for Δ*z*_l_ = 1200 nm.

### 3.4. Increased energy band width

**Figure 8:**
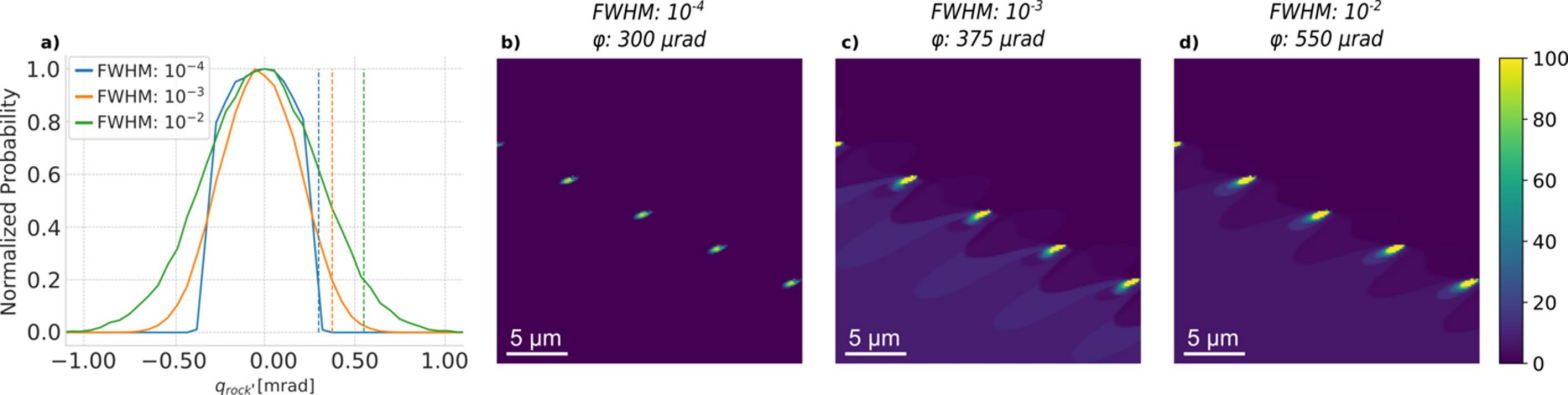
(*a*) Normalized distributions of the reciprocal-space resolution function in the 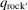 direction at different energy band widths (FWHM). (*b*) A simulated image with an energy band width of 1.41 × 10^−4^ at 300 µrad in the rocking curve, blue dashed line in panel (*a*). (*c*) A simulated image with an energy band width of 1.41 × 10^−3^ at 375 µrad in the rocking curve, orange dashed line in panel (*a*). (*d*) A simulated image with an energy band width of 1.41 × 10^−2^ at 550 µrad in the rocking curve, green dashed line in panel (*a*). The DFXM images have the same exposure time and colour scale for direct comparison, with maximum intensities of 100, 815 and 1806 in panels (*b*)–(*d*), respectively.

The resolution function in the direction of a rocking scan is illustrated in Fig. 8 ▸(*a*) as a function of the energy band width (assuming a Gaussian energy distribution and a fixed divergence of the incident beam). The resolution function is seen to be almost identical for band widths of 10^−4^ and 10^−3^, a reflection of the fact that the reciprocal-space resolution functions for these values are dominated by the incoming divergence. In contrast, the resolution function becomes approximately 50% wider when changing the band width from 10^−4^ to 10^−2^.

The corresponding changes in weak beam contrast are shown in Figs. 8 ▸(*b*)–8 ▸(*d*). Here, increases in incident intensity of factors of 10 and 100 for 10^−3^ and 10^−2^, respectively, have been taken into account (this normalization is described in Appendix *B* [App appb]). Note that all three images represent ϕ values where the intensity far away from the dislocations has dropped to 20% of its maximum value [as illustrated by the three vertical lines in Fig. 8 ▸(*a*)]. Two effects are evident. Firstly, for the 10^−2^ case one needs to go to larger ϕ to obtain the same weak beam contrast. Secondly, the increased incident flux improves the counting statistics significantly in both cases.

### 3.5. Validation of COM map for visualization of dislocations

**Figure 9:**
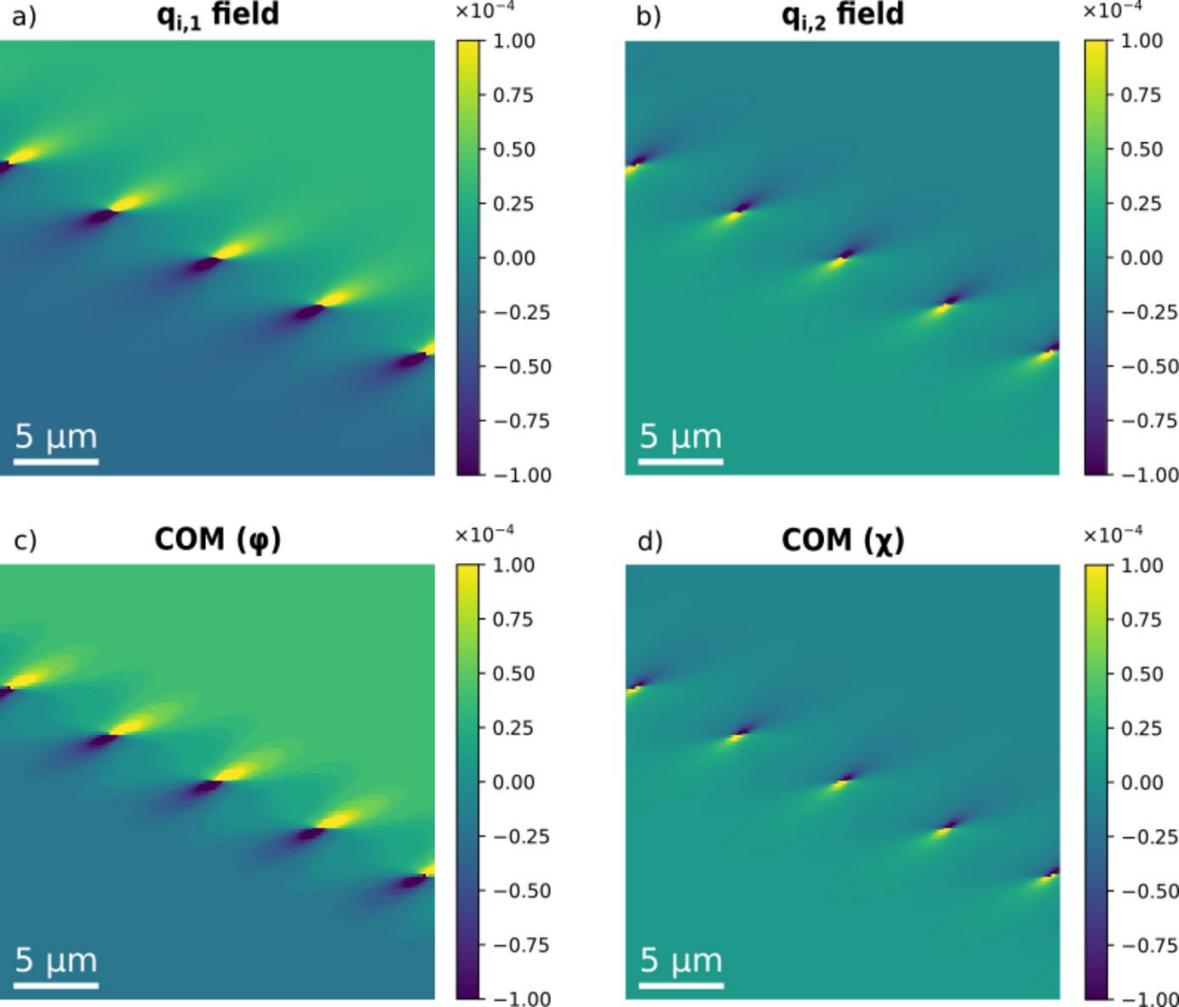
A test of the accuracy of the centre-of-mass map concept based on a (ϕ, χ) scan and using the same phantom as in Fig. 4. (*a*) The model **q**_i,1_ field and (*c*) the corresponding **q**_i,1_ field derived from the COM mapping of DFXM images. (*b*) The model **q**_i,2_ field and (*d*) the corresponding **q**_i,2_ field derived from the COM mapping of DFXM images.

The phantom presented in Fig. 2 ▸ (with *g* = 4 µm) can be used as a test of the appropriateness of the heuristic, the COM map as defined in Section 2.7. Specifically, we consider a mosaicity scan. Shown in Fig. 9 ▸ is a comparison of the modelled and retrieved versions of the two **q** components. The correspondence is excellent. Additional simulations show that regions closer to the core (with strains of magnitude up to 5 × 10^−4^) are reproduced equally well, provided the ϕ and χ ranges are sufficiently wide to include the tails of the distributions. The method also handles models with a larger vertical beam height well, *e.g.* the phantoms used in Fig. 7 ▸.

### 3.6. Contrast in a dislocation wall

The specified dislocation configuration is well known to give rise to a domain wall, separating two domains that are rotated by an angle α = *b*/*g* with respect to each other around an axis parallel to the dislocation line. Here *b* is the length of the Burgers vector and *g* is the distance between neighbouring dislocations in the dislocation coordinate system.

**Figure 10:**
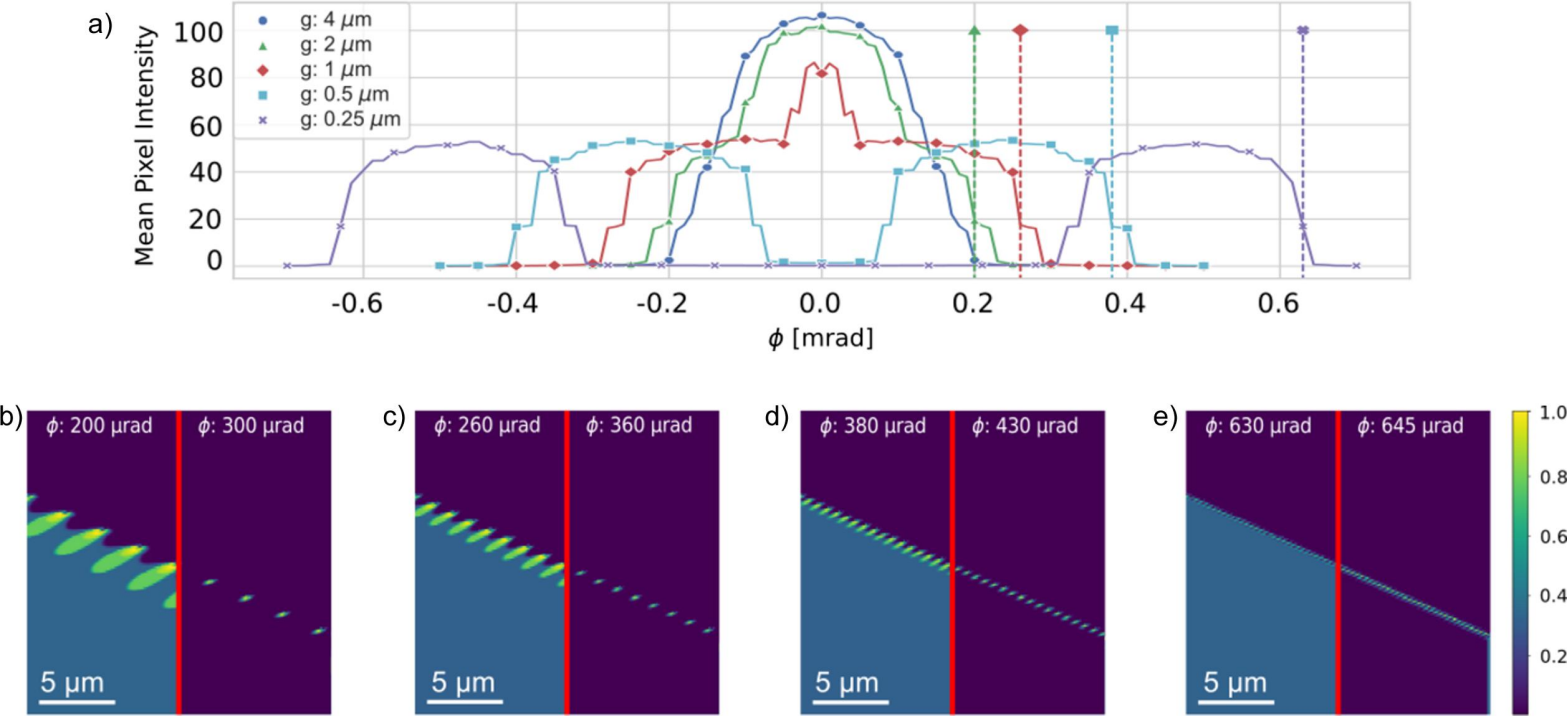
Contrast in the domain wall. Forward simulations of the rocking curve while varying the distance *g* between neighbouring dislocations: *g* = 2, 1, 0.5 and 0.25 µm. (*a*) The integrated intensity across the entire image as a function of rocking angle ϕ. With decreasing *g* the initial strong beam region around ϕ = 0 splits into two separate regions, corresponding to the two domains separated by the wall. Positions where the integrated intensity has dropped to 20% are defined by dashed lines. The corresponding DFXM images are shown in the left-hand parts of panels (*b*) *g* = 2 µm, (*c*) *g* = 1 µm, (*d*) *g* = 0.5 µm and (*e*) *g* = 0.25 µm, respectively. Shown in the right-hand parts of the same panels are images at the tail of the intensity distribution, in ‘extreme weak beam conditions’. The ϕ values are indicated. Corresponding DFXM images for *g* = 4 µm are provided in Fig. 4.

In Fig. 10 ▸ the contrast in and around the wall is shown as a function of *g*. It appears that, with decreasing *g*, the initial strong beam region around ϕ = 0 splits into two strong beam regions, corresponding to a domain in the upper right-hand part of the DFXM images (with contrast at negative ϕ values) and another domain in the lower left-hand part (with contrast at positive ϕ values). At *g* = 0.25 µm, the misorientation between the domains is α = 960 ± 10 µrad, when calculated as the distance between the centres of the two peaks of the magenta curve in Fig. 10 ▸(*a*). In comparison, α = *b*/*g* gives α = 1.14 µrad for the same spacing. The lower value obtained in the DFXM data is due to not probing the full misorientation. Only the rotation component around the *y*_l_ axis is probed. Splitting of the misorientation into a rotation around the *y*_l_ axis and a subsequent rotation without a component around this axis yields a rotation angle around *y*_l_ of α = 954 µrad. The correspondence is a testimony to the high angular resolution of DFXM. As illustrated in Figs. 10 ▸(*a*)–10 ▸(*d*) the individual dislocations are still visible, provided one changes the ϕ setting to keep the relative position to the strong beam condition. At a given spatial resolution the field becomes more and more confined around the core of the dislocations. This is a well known effect of the superposition of the fields from the individual dislocations for such walls. Eventually, with decreasing *g*, contrast is lost due to limitations in spatial resolution.

## 4. Discussion

### 4.1. Applications of geometrical optics simulations

In this paper, for reasons of simplicity, the micromechanical model used in the forward simulation is a simple superposition of the analytical expressions for individual dislocations. However, the simulation tool is generally applicable to elastic or viscoplastic models, *e.g.* in connection with phase-field simulations, discrete dislocation dynamics (DDD; Pachaury *et al.*, 2022 ▸) or continuum dislocation dynamics (CDD; El-Azab & Po, 2020 ▸) models. In particular, the forward projection will support the ambition outlined by Poulsen (2020 ▸) of acquiring an experimental 3D movie of the dislocations and using the configuration of the first time step as the initial configuration for a corresponding 3D movie based on simulations. The two movies can then be compared at tens of millions of points in space and time, providing a very thorough test of the validity of the micromechanical model.

In this context, it is an asset that the geometrical optics code is fast. The code previously detailed by Poulsen *et al.* (2021 ▸) has been reimagined in Python and extensively optimized, resulting in significant enhancements in computation efficiency. It is now executable on a standard laptop equipped with an Intel 11th-generation i7 processor and no GPU, facilitating swift execution of both the reciprocal-space component (probing 10^8^ rays for MC simulations) and the direct-space element of the resolution function (35 million data points). The former is completed within 45 s, while the latter is accomplished in less than 3 s. By incorporating additional vectorization and parallelization techniques or harnessing the superior capabilities of readily accessible server hardware and GPUs, the simulations can be further fine-tuned to achieve optimal performance. Hence, with optimization of the code it will be possible to fit material parameters by simulating a large set of forward projected images corresponding to different parameter values, and to identify the parameter where the corresponding image resembles the experimental one the most. [A similar fitting procedure was demonstrated in a grain growth study by Zhang *et al.* (2018 ▸) based on DCT data. There, the comparison of the experimental 3D movie with phase-field simulations led to the simultaneous determination of 5000 unknown material parameters in the case of reduced mobilities.]

The fast generation of images also suggests another application of the code: for the generation of large sets of images to be used for training neural networks, *e.g.* in connection with the identification of Burgers vectors and slip systems (Huang *et al.*, 2023 ▸).

The simulations can be repeated for any number of diffraction vectors such that all components of the displacement gradient tensor field are encoded in the images. In this paper we have only considered the line beam modality of DFXM. The geometrical optics code also works without adaptation for an impinging box beam – implying a projection geometry – and with a minimum of adaptation for most other modalities, *e.g.* where apertures are inserted in the back focal plane.

### 4.2. Applications of wavefront propagation software

The relevance of using this code for studies of the effects of coherence and dynamical diffraction is obvious. The results shown in Fig. 4 ▸ reveal that these effects are not detrimental for visualizing dislocations. In particular, for weak beam scattering the kinematical approximation is well justified.

The code has been verified by comparison with experimen­tal data for stacking faults in diamond (Carlsen *et al.*, 2022 ▸).

### 4.3. Applications of COM maps and FWHM maps

The aim of the COM map is to condense the information on strain components embedded in a set of (experimental or simulated) images into one map, with a ‘best estimate’ of the strain field in each voxel. For 2D or 3D scans such as (ϕ, χ) or (ϕ, χ, 2θ) scans the COM map is a vector map. In particular for the higher-dimensional scans, generation of this map reduces both memory and time demands and eases visualization. Optimization of micromechanical models can then potentially be performed, based on the resemblance of corresponding experimental and simulated COM maps. However, experimental COM maps are currently, to varying degrees, limited by coherence and dynamical diffraction effects. In principle, methods similar to *e.g.* high-angle annular dark-field TEM can be applied to reduce such effects. The DFXM community is currently working on more heuristic methods, including the use of machine learning. The COM map complements the association of each voxel with its COM (ϕ, χ) values, as calculated *e.g.* by *darfix*, as the software produces the exact same maps for the forward simulated images from geometrical optics.

In a similar manner, the FWHM map is introduced to provide a simple way of measuring the strain variation within each voxel. For higher-dimensional scans it will generate a vector field. This variation will be a function of the dislocation density. It can be seen as a rather primitive but fast way of generalizing the concept of line broadening, well known from X-ray powder diffraction (Ungar *et al.*, 1984 ▸) and grain mapping (Nisr *et al.*, 2012 ▸), to voxel-based mapping. Again the FWHM maps condense information from many images into one, and the direct comparison of experimental and simulated FWHM movies may improve plasticity models.

### 4.4. Implications for experimental setups

The results for the simple phantoms presented above lead to several insights and hints for data analysis. In random order, we note the following:

(i) *Strong and weak beams.* This work underlines the opportunities and challenges given by the anisotropic reciprocal-space resolution function. According to equations (19)–(21) in the work of Poulsen *et al.* (2017 ▸) we have the following analytical expressions for the width in the three directions (FHWM): 

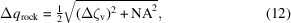




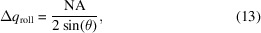




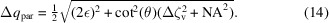

Inserting values for the settings used here into equations (12), (13) and (14), we get the following strain sensitivities, defined by the HWHM, in the rocking, rolling and 2θ directions, respectively: ±1.95 × 10^−4^, ±1.17 × 10^−3^ and ±0.49 × 10^−3^. These numbers are in good agreement with the widths in Δϕ, Δχ and 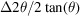 , respectively, as observed in the figures in this paper.

This work also indicates that one may define optimal settings, say for weak beam contrast, in terms of positions where the intensity of the undeformed ‘matrix’ is reduced to a certain percentage, such as 20%.

(ii) *Vertical beam height.* For most of the reported DFXM studies from the ESRF, the beam height is 600 nm (Yildirim *et al.*, 2023 ▸). An analysis of simulated DFXM images shows that the angle of the associated streak with the laboratory axis can be found within ±1°. For well separated dislocations this information can be used to infer the direction of the dis­location line (*e.g.* one gets 3D information despite each image being only 2D), which in turn can be used for identification of the active slip systems. However, for complete identification of the type of dislocation, the variation in position in *z* must be included and/or a parameterization of the surrounding field.

(iii) *Use at an XFEL.* Within the last couple of years DFXM studies have been performed at several XFELs (Dresselhaus-Marais *et al.*, 2023 ▸). The X-rays emerging from the self-amplified spontaneous emission process at XFELs have an intrinsic energy band width of 10^−3^. Everything else being equal, Fig. 8 ▸ suggests that this increase in band width will have a positive influence on contrast. In practice, pre-seeding or monochromators are often used. Quantitative mapping of tensor fields using XFEL data is challenging due to coherence effects and is likely to require more advanced simulation tools. However, for some studies the geometrical optics code may be relevant. This is corroborated by the recent simulations and subsequent experimental DFXM demonstrations of phonon photography: the visualization of acoustic waves in bulk specimens with sub-picosecond resolution (Holstad *et al.*, 2022 ▸, 2023 ▸).

(iv) *Use of a pink beam.* At the time of writing, a fully dedicated DFXM beamline, ID03, is becoming ready for commissioning at the ESRF. This will provide the option of using a pink beam with an energy band width of 10^−2^. The pink beam will radically increase the likelihood of radiation damage, and it will increase the vertical beam height as the CRL optics are chromatic. Nevertheless, the factor of 100 in increased brightness may be favourable for many DFXM studies of domains as the data acquisition can be faster. For dislocations, one may worry that the rocking scan contrast is lost, due to the resolution function in the ϕ direction becoming larger than or equal to the strains within our resolution capability. This can for some purposes be overcome by noting that the centre positions of a peak can be determined to a precision that is much better than the width of the same peak. However, as shown in Fig. 8 ▸, the signal-to-noise ratio in the weak beam contrast is improved, despite the fact that images need to be acquired at larger absolute ϕ values.

(v) *A future optics upgrade.* This will involve the introduction of multilayer Laue lenses (MLLs) to replace the CRL optics (Bajt *et al.*, 2018 ▸; Murray *et al.*, 2019 ▸). MLLs have been demonstrated to exhibit an NA that is five times that of existing CRLs. This, in combination with high manufacturing accuracy, implies that the incoming beam height and the resolution function in the sample plane can both be improved by a factor of five. With this in mind, we can now apply the geometrical optics code to explore the effect of a larger NA on weak beam contrast.

## 5. Conclusions

Forward simulations by means of (kinematical) geometrical optics expressions with full inclusion of the direct-space and reciprocal-space resolution functions have been shown to be a powerful tool for DFXM studies of dislocations. The comparison with wavefront propagation validates the approach in the weak beam limit. The simulations are fast and versatile and may apply to a range of tasks, including:

(i) Optimization of experimental setups, training, and exploratory studies of new DFXM modalities.

(ii) Guidance, optimization and validation of micromechanical models such as DDD and CDD models by direct comparison of simulated and experimental movies.

(iii) Creation of training sets for classification by means of supervised learning by neural networks.

Varying the experimental parameters shows that the singularity in the strain field generally favours the visibility of isolated dislocations provided they can be resolved spatially. The main limiting factors for DFXM studies of individual dislocations are therefore the dislocation density and the spatial resolution.

For orientation mapping of grains and domains, the variations in orientation are typically larger than the NA of the objective and the reciprocal-space resolution function can in general be neglected. For such tasks, it is well known that the orientation of each voxel is well described by the COM of local orientations. We have demonstrated here that in the idealized setting (based on the kinematical limit) the COM map can also be used to quantify and visualize the field of strain components around dislocations. The relevance of this map for experimental data – which may be influenced by dynamical diffraction effects – needs careful investigation.

In order to provide full transparency and facilitate reproducibility, we have made available as external supplementary information all the source code and implementation details used to generate these simulations. For the geometrical optics simulations, see https://doi.org/10.5281/zenodo.8370255. For the wavefront propagation simulations, see https://doi.org/10.5281/zenodo.8370309.

## Supplementary Material

Source code and implementation details: geometrical optics simulations: https://doi.org/10.5281/zenodo.8370255

Source code and implementation details: wavefront propagation simulations: https://doi.org/10.5281/zenodo.8370309

## Figures and Tables

**Table 1:** Monte Carlo simulations to provide an absolute scale for the simulations performed in Section 3.4

Energy band width (FWHM)	1.4 × 10^−4^	1.4 × 10^−3^	1.4 × 10^−2^
Relative incident intensity	1	10	100
Transmission (%)	30	26	5.3
Normalized intensity	1	8.6	18

## Appendix A Deformation field around a single edge dislocation

For reference, the displacement field **u**_d_ = (*u*_d,*x*_, *u*_d,*y*_, *u*_d,*z*_) around a single edge dislocation with the dislocation line intersecting the origin, the Burgers vector along the *x* axis and the line pointing along the *z* axis is defined by Hirth & Lothe (1992 ▸) as

where *b* is the length of the Burgers vector and ν is the Poisson ratio. In the present example we consider aluminium at elevated temperature, with *b* = 3.507 Å and ν = 0.334. The corresponding non-zero components of the deformation gradient tensor in the dislocation system **F** ^d^ are

The corresponding **H** tensor field as defined by Poulsen *et al.* (2021 ▸) and the elastic distortion tensor  are

## Appendix B Incorporating the incident flux in the geometrical optics simulations

The Monte Carlo code for simulating Res_*q*_ provides a normalized distribution; it is not well suited to comparing intensities in DFXM images across variations in incident beam parameters such as changes in the energy band width. To facilitate the comparison in Section 3.4 we scaled the simulations using estimates for the DFXM strong beam intensity provided by an auxiliary Monte Carlo ray-tracing program. For each ray the exit beam direction is given by the Laue condition and subsequently one can deduce the probability of the diffracted beam being transmitted through the objective.

The results of these simulations are summarized in Table 1 ▸.
